# Substance Abusers in an Acute Psychiatric Facility: A Diagnostic and Logistic Challenge

**DOI:** 10.1155/2013/705657

**Published:** 2013-02-21

**Authors:** John E. Berg, Asbjørn Restan

**Affiliations:** ^1^Oslo and Akershus University College, Faculty of Health Sciences, PB 4, St. Olavs Plass, 0130 Oslo, Norway; ^2^Blakstad Psychiatric Hospital, Vestre Viken Health Trust, Vettre, Norway

## Abstract

Acute resident psychiatric facilities in Norway usually get their patients after referral from a medical doctor. Acute psychiatric wards are the only places accepting persons in need of emergency hospitalisation when emergency units in somatic hospitals do not accept the patient. Resident patients at one random chosen day were scrutinized in an acute psychiatric facility with 36 beds serving a catchment area of 165 000. Twenty-five patients were resident in the facility at that particular day. Eight of 25 resident patients (32.0%) in the acute wards were referred for a substance-induced psychosis (SIP). Another patient may also have had a SIP, but the differential diagnostic work was not finished. A main primary diagnosis of substance use was given in the medical reports in only 12.9% of patients during the last year. Given that the chosen day was representative of the year, a majority of patients with substance abuse problems were given other diagnoses. There seems to be a reluctance to declare the primary reason for an acute stay in a third of resident stays. Lack of specialized emergency detoxification facilities may have contributed to the results.

## 1. Introduction

Acute treatment of substance abuse is handled differently from country to country. The abusers may be referred to a somatic hospital, taken care of in police custody, by specialized detoxification centres or within the realms of an acute psychiatric hospital. Referral from a medical doctor is the main route to a resident stay in an acute psychiatric facility in Norway. Patients with substance abuse problems often have symptoms as dramatic as the mainstream patients referred to an acute psychiatric facility, even when comorbid psychiatric diseases in the substance abusers are lacking [1]. Health and social workers contemplating referral of a person with acute stress or severe suicidal or violent behaviour may have difficulties differentiating between substance abuse and psychiatric morbidity.

Substance-induced suicidal admissions to an acute psychiatric facility may be frequent as shown in a study from the USA [2]. Such patients have a high degree of addiction severity with only temporary substance-induced suicidal ideas. They are automatically offered highly qualified and expensive services even if not in need of them. The authors argue that psychiatric inpatient services should provide specific and intensive addiction intervention treatment or that the outpatient addiction services/relevant addiction detoxification units could be able to provide such emergency inpatient services even for suicidal patients. The mix of these two patient populations in the same psychiatric facility may not be easy to handle, or advantageous to the incumbents [3, 4]. On the other hand the probability, of a repetition of a sucidal attempt at discharge must be thoroughly evaluated as shown in a recent study from Italy [5].

Alcohol-abusing patients, when referred to an acute psychiatric ward for suspected delirium, may develop a Korsakoff syndrome after many years of abuse. All such patients must get high-dose parenteral thiamine (100–200 mg IM/day) wherever they are admitted to reduce the probability of developing a psychosis that may be difficult to treat [6]. This may perfectly well be administered outside an emergency ward for general medicine or psychiatry. 

Patients who have substance-use-related emergency admission to a general hospital are at increased risk of readmission [7]. A substantial proportion of them also have contacts with outpatient psychiatric services. 

Self-injurious behaviour has been shown to have a lifetime prevalence rate of 49% in a group of opiate addicts admitted to community and inpatient treatment programmes [8]. Such behaviour may, when viewed through purely psychiatric lenses, be interpreted as a symptom and part of a psychiatric illness, rather than as part of the substance abuse. 

Symptom patterns in the week prior to admissions to an emergency psychiatric facility for suicide attempt were examined in 1547 Italian patients [9]. The following factors were positively related to attempted suicide: substance abuse, nonprescribed medication abuse, and depressive symptoms. Alcohol abuse did not attain statistical significance. 

Fifteen per cent of 5641 patients admitted to an adult emergency department for medical care in a large, inner-city hospital had alcohol or drug dependence [10]. The comorbidity was a challenge for finding correct treatment. 

Thus it would be of interest to study the referrals to an emergency psychiatric ward in a country where emergency detoxification is not offered in specialized facilities. 

## 2. Materials

Resident patients at one random chosen day were scrutinized in an acute psychiatric facility with 36 beds serving a catchment area of 165 000. Twenty-five patients were resident in the facility at that particular day. Mean age was 44.0 with range from 21 to 84. Only one author (J. E. Berg) had access to the medical file of the patients as a senior consultant in the wards, and summary of patients of Table 1 was done anonymously. 

Substance abusers have access to a separate line of treatment and rehabilitation facilities, but these do not accept emergency admissions. When deemed necessary, emergency treatment of substance abusers is defined either as a medical emergency (alcohol delirium, respiratory distress) or as a case of severe suicidality or psychotic behaviour. In the former case the person is referred to a medical emergency ward and in the latter to a psychiatric emergency ward. 

## 3. Results

Eight of 25 resident patients (32.0%) in the acute wards were referred for a substance-induced psychosis (SIP). None of them developed a prolonged psychotic illness. Another patient may also have had a SIP, but the differential diagnostic work was not finished when closing data collection. 

During the last full year 807, patients were referred, 433 females and 374 males.  Table 2 shows the distribution of main diagnoses. Substance abuse, defined as diagnosis group ICD-10 10–19 contributed 12.9% of patients. Fifty-eight per cent of patients (58.0%) had psychotic and affective diagnoses. 

## 4. Discussion

A random day summary of treatment relevant diagnoses showed that 32.0% of patients in an acute facility had substance-induced psychosis. During one year 12.9% of resident patients were given a substance abuse diagnosis. There seems to be an unwillingness to report the presence of even severe substance abuse as long as the patient has F20, F30, or F60 group diagnoses, as reported from the facility of the present study two years ago [11].

Amaral et al. found that 28% of all patients in medical emergency units had substance use disorders, but less than half of the patients with alcohol-related problems were identified [12]. 

The usefulness of brief intervention for patients with acute alcohol intoxication in emergency medical services has already been shown [13]. A liaison group consisting of a psychiatrist, a nurse, and a psychologist with special training in addiction medicine performed intensive case management after a preset protocol. They compared a group of 106 patients with a control group of 97 getting treatment as usual. In the control group 59% of the patients were readmitted for the same reason within 1-year follow-up against 32% in the intervention group. In our opinion, it could be possible to implement such an intervention within an acute psychiatric department. The professions of the liaison group in the cited study are regularly present in psychiatric department(s) of general hospitals. In our opinion, organising alcohol abuse treatment in special clinics within the psychiatric hospital could actually hamper, rather than promote further change in such patients. It would probably also be far more expensive and less cost effective. 

Organization of emergency admissions for substance abusers could be other than what is the case in Norway. One possibility is the already cited model from Italy. This model was, however, established to ameliorate the dramatic consequences for Italian psychiatric in-patient services after the Legge 180 change in the 1980s when all asylums were closed. Another possibility would be to establish specialized emergency wards within the substance abuse treatment chain. Our study indicates that this, combined with diagnostic discipline, might lead to several desired results.

1. Psychiatric emergency facilities might concentrate on their primary functions and thus become clinically more efficient, and even more cost efficient.
2. A similar development should be observed within the substance abuse treatment chain.
3. The Italian liaison model might, probably at low cost, be served by a team from either the specialized substance abuse ward or from the acute psychiatric facility in those cases where somatic considerations render a medical emergency ward the only feasible option.

A weakness of the present study was that we had no access to secondary diagnoses on the patients during the year. Clinical experience indicates that substance abuse is often relegated to lower ranks on the list of diagnoses given to the single patient. The task of leafing through all medical records to find all relevant diagnoses was not undertaken for the present study. Another weakness could be the choice of just one day for calculating the number of substance abusers in the ward. 

The on-going discussion among clinicians whether people may have a substance abuse diagnosis without also satisfying other axis I diagnoses is not settled, despite the results of the seminal work of Regier et al. and Davies [1, 14].

Combining the handling of severe psychiatric illness and substance abuse in the same ward may be challenging, as indicated, for instance, by the difficulties encountered with detoxification of benzodiazepine abusers within the acute psychiatric ward [3]. Awareness of the different approaches needed is a salient task for nurses and doctors alike. 

## Figures and Tables

**Table 1:** Characteristics of resident patients in an acute department on one random chosen day according to sex, type of abuse, presence of suicidality at admission, diagnosis, use of compulsory admission and/or compulsory treatment with medication according to the Norwegian Law, and the development of substance induced psychosis (W: women, M: men).

	Sex/age	Abuse	Suicidality	ICD-10	Coercion	Comment
1	w/51	Alc/H/B	None	F31	Yes	SIP
2	w/30	None	None	F31	Yes	—
3	w/51	Alcohol	Yes	F32	No	15 AU/day
4	w/51	Alcohol	None	F32	No	Detox-wish, member of Alc-Anon
5	w/62	None	None	F25	Yes	Neuroleptic malignant syndrome
6	w/23	Cannabis/alcohol	None	F20	Yes	Substances not relevant this time
7	m/23	Cannabis/alc/MDMA	Yes	F31	Yes	SIP/bipolar mania
8	w/55	None	None	F22	Yes	—
9	w/51	Alcohol < 5 AU/day	None	F31	Yes	—
10	m/57	Alcohol < 5 AU/day	Yes	F60	No	ECT for depression
11	k/43	Heroin/amphetamine	Yes	F25	Yes	Substance use only in suicidal attempts
12	m/21	Cannabis/cocaine	None	F12/F14	Yes	SIP
13	m/84	None	None	F00	No	—
14	k/60	None	None	F31	No	—
15	m/23	None	None	F31	Yes	—
16	m/42	Alcohol/amphetamine	None	F10/F15	Yes	SIP
17	k/45	None	Yes	F32	No	—
18	k/46	Alc/H/B/MDMA/Can	None	F20	Yes	SIP
19	k/58	Alcohol	Yes	F10	Yes	SIP
20	w/35	None	Yes	F41	No	—
21	w/36	None	Yes	F20	Yes	—
22	w/20	None	Yes	F43/F60	Yes	—
23	w/62	None	Yes	F42	No	B, previous overuse
24	w/26	Cocaine/cannabis	None	F14/F12	Yes	SIP?
25	m/45	None	Yes	F32/F31?	No	—

SIP: Substance-induced psychosis.

Alc: Alcohol, AU: Alcohol Unit = 12 g pure alcohol = 0.33l beer = 1 glass of wine.

Alc-Anon: Alcoholics Anonymous group.

H: Heroin or other opioid substance, including methadone and buprenorphine.

B: Benzodiazepines, MDMA = (3,4-Methylenedioxymethamphetamine) or ecstasy.

ECT: Electroconvulsive treatment.

F31: bipolar disorder; F32: depressive episode; F25: schizoaffective disorder; F20: paranoid schizophrenia; F22: delusional disorder; F60: personality disorder; F10–19: substance abuse disorders; F00: dementia; F41–43: anxiety, compulsive and related disorders.

**Table 2:** Medical record derived main diagnosis in a one-year cohort of patients in an acute psychiatric facility.

ICD-10 diagnostic group	Total	Male	Female
F0	13	5	8
F10	104	59	45
F20	247	138	109
F30	221	102	119
F40	88	35	53
F50	3	1	2
F60	65	6	59
F70	4	1	3
F80	5	3	2
F90	15	9	6
Other diagnoses	42	15	27

Sum	807	374	433
